# The Effect of Magnesium Deficiency on Neurological Disorders: A Narrative Review Article

**Published:** 2019-03

**Authors:** Wenwen XUE, Jing YOU, Yingchao SU, Qinglu WANG

**Affiliations:** 1. Key Laboratory of Biomedical Engineering & Technology, Qilu Medical University, Zibo, China; 2. Department of Biomedical Engineering, College of Engineering, University of North Texas, TX, USA; 3. College of Life Science, Shandong Normal University, Jinan, China

**Keywords:** Magnesium, Neurological disorders, Alzheimer’s disease, Parkinson’s disease, Stroke, Migraine

## Abstract

**Background:**

Magnesium (Mg) is an essential element for the body. It is a cofactor for ATP, DNA, and RNA and more than 600 enzymes. As it is similar to Ca^2+^, this element can also act as a cell signaling molecule and play multiple important roles in the nervous, muscle, and immune systems. Recent studies have associated Mg-deficiency with many neurological disorders, such as cerebral vasospasm, Alzheimer’s disease, stroke, and migraine. As it plays such a crucial role in human body, therefore, we summarized the role of Mg in neurological disorders to illustrate the symptoms caused by Mg-deficiency and the possible underlying mechanisms.

**Methods:**

We critically discuss the role of it that we review the recent literature of magnesium. We also review the available data which are concerning the role of magnesium in neurological disorders.

**Results:**

Magnesium is related to neurological disorders on the basis of the study of animals and humans experiments. Furthermore, these nervous systems related diseases include cerebral vasospasm, Alzheimer’s disease, Parkinson’s disease, stroke and migraine.

**Conclusion:**

Magnesium has effects on neurological disorders, such as its utility in cerebral vasospasm, Alzheimer’s disease, Parkinson’s disease, stroke and migraine. So here we make a brief review to conclude it.

## Introduction

Magnesium (Mg) is the second most abundant intracellular cation and the fourth most abundant element in the body (1). Mg plays a complex function in our body but accounts for only 0.05% of body weight. This element is regarded as the macro element in the human body and important for human health (2). As an activator of many enzymes, Mg is involved in the metabolism and neurotransmission of the three major nutrients in the body. Acute or chronic Mg deficiency may affect the nervous system. Acute Mg deficiency leads to metabolic encephalopathy and alteration of neuromuscular excitability, such as deprementia and nervousness. By contrast, chronic Mg deficiency is characterized by spasm. Although the potential role of Mg in neurological diseases has been established for decades, the clinical arena of translation is difficult to isolate (3). Mg deficiency leads to neurological disorders ranging from apathy to psychosis. Moreover, Mg has an effect on the regulation of synaptic plasticity (4).

Several studies have suggested a neuroprotective action of Mg in the synaptic function (5). The results from an in vitro study with mouse neurons showed that Mg involved in N-methyl-D-aspartate (NMDA)-receptor and inhibited the release of glutamate (6,7). Thus, given such critical function, we summarize briefly the role of Mg in neurological disorders.

### Role and sources of Mg

Mg is a co-factor for more than 300 enzymes and a key nutritional mineral to regulate numerous biochemical reactions (8). Thus, this element is vital for numerous physiological, cellular, and biochemical functions (9). In living cells, Mg is involved in the homeostasis of other minerals, such as sodium, potassium, and calcium, as well as in the formation, transfer, storage, and utilization of adenosine triphosphate (ATP), a principal source of energy. In the human body, Mg is involved in the maintenance of normal muscle and nerve function, heart rhythm, bone strength, and immune system. Consequently, daily proper in-take of this element is required. The recommended dosage of Mg for adults is 300–420 mg/day (10). Mg can be obtained from all kinds of foods and water. Mg-rich foods include grains, nuts, vegetables, and fruits. Actual intake of Mg is determined by various factors. Moreover, the amount of Mg from the same source may vary (Table 1). For example, the amount of Mg in water from different brands may differ (ranging from 1 mg/L to more than 120 mg/L) (11).

**Table 1:** Part Sources of Magnesium

***Class***	***Food***	***Milligrams(mg)per serving(3.5oz)***	Percent DV*
Cereal	Oat	177	50
	Wheat	126	35
	Corn	37	10
Bean	Soybean	280	79
	Black bean	160	45
Nut	Cashew	292	82
	Almonds	268	75
	Pine nut	251	71
	Peanuts	184	52
	Hazel	163	46
	Walnut	158	45
	Macadamia nut	130	37
Vegetables	Spinach	79	22
	Parsley	50	14
	Pea	33	9
	Garlic	25	7
	Broccoli	21	6
	Coconut	32	9
Fruits	Avocado	29	8
	Banana	27	8
	Papaya	21	6
	Blackberry	20	6
Meat	Pork	28	8
	Mutton	23	6
	Beef	21	6
Fungoid	Enoki Mushroom	16	5
	Egg	10	3
	Milk	10	3

* DV = Daily Value. DVs were developed by the U.S. Food and Drug Administration (FDA)

### Neurological disorders

Solid neuroscience has associated neurological disorders with Mg (12). Damage to the peripheral nerve will result in various types of paresthesia and neurodegenerative diseases [e.g., cerebral vasospasm, stroke, Alzheimer’s disease (AD), and migraine]. Chronic neurological disorders will lead to a serious condition which will result in permanent nerve damage causing irreversible brain loss. Neurological disorders have been escalating as a public health problem. These disorders will not only lead to destructive damage in the patient himself by suffering from pain and reduced quality of life, but the family and society will also undertake a heavy load. Neurological disorders are caused by various pathogenesis. Several trials, such as enzyme replacement therapy and gene therapy for nervous system diseases to release the pain, have been conducted. However, these trials do not improve the conditioner alter the final outcome of the disease. Thus, the neurologic disease is still seldom curable. The neuroprotective role of Mg has been proved, but the role of Mg in pathogenesis remains ambiguous. Using a carrier, such as polyethylene glycol, can solve this problem while reducing the dose of Mg on central effects and the deleterious peripheral effects (3).

### Mg deficiency and neurological symptoms

Mg is an essential ion in almost all living cells (13) and displays numerous intracellular physiological functions. Thus, Mg deficiency has been associated with numerous clinical disorders worldwide. Abnormality in Mg metabolism caused by Mg deficiency also affects other electrolyte and enzymatic activities. Therefore, compared with hypermagnesemia, hypomagnesemia, which is an Mg imbalance, is more likely to lead to unnecessary nerve and muscular excitation hyperfunction (14), manifested as muscle tremor, tetany, hyper-reflexia, dystaxia, delirium, neurological disorders, and convulsion in severe cases. Major manifestations and causes of magnesium deficiency in neurological diseases are summarized (Table 2).

**Table 2:** Major manifestations and causes of disease

***Disease***	***Major manifestations (Mg deficiency) ***	***Causes (Mg deficiency)***
Cerebral vasospasm	Acute focal vasospasm Contraction reaction Platelet aggregation	Insufficient magnesium intake Excessive loss of digestive tract (Deleterious effect of common medications on magnesium absorption) ( 60 ) Excessive loss of kidney (61) Hyperthyroidism
Stroke	Inflammatory response Oxidative response	
AD	Reduced synaptic plasticity	
PD	α-synuclein aggregation Release of neurotransmitter	
Migraine	Hyper aggregated of platelets Cortical spreading depression	

### Mg deficiency and cerebral vasospasm

Cerebral vasospasm is the persistent contraction of the intracranial artery, and this condition has no typical clinical symptoms (15). The pathogenesis of this disorder is still unclear. Considering that in the cerebral arteries, the contractile response to norepinephrine is enhanced in the decreased Mg^2+^, and the delayed response is unchanged, in previous study have already proved that the role of Mg^2+^ deficiency in the development of cerebral vasospasm (16). Hypomagnesemia is associated with acute focal vasospasm in the coronary arteries (17). Mg plays a key role in the regulation of the excitability of cell membranes. This element antagonizes the NMDA receptor (2, 13) on the cell surface and intracellular voltage-gated calcium channels (13, 18). Thus, calcium entry to ischemic neurons, which is crucial for the activation of cellular apoptotic pathways, is impeded. Mg is a neuroprotective agent in different models of cerebral ischemia (19). Hence, Mg deficiency should be considered as a cause of various neurological symptoms. Cerebral vasospasm is mainly diagnosed during the deterioration of the nervous system. Prompt neuroprotective treatment is needed to prevent clinical defects (20). Highly permeable Mg salt can be used in the neuroprotective treatments for cerebral ischemia. Although experimental evidence has supported theoretical investigations, additional studies with strong evidence should be conducted (21). Injecting MgSO_4_ reduced the incidence of cerebral vasospasm. The action of Mg, namely, vasodilatation, inhibition of free radical formation, impedance of vasoconstrictive substances, and inhibition of platelet aggregation, resulted in the remission of cerebral vasospasm (22). No other study has reported on cerebral vasospasm related to hypomagnesaemia. The symptoms of this neurological disorder will be completely resolved with the rapid detection and correction of serum Mg levels. Further studies are needed to confirm these hypotheses.

### Mg deficiency and stroke

Stroke is the cerebral blood circulation disorder which results in loss of local neurological function (23). This disorder is the second leading cause of death and a leading cause of adult disability worldwide. Although the incidence of stroke has been declining in developed countries, this condition continues to increase worldwide because of the aging society. Nevertheless, current therapies for acute ischemic stroke are reper-fusion-based and only moderately effective (24, 25). Mg treatments exhibited neuroprotection in some disorders, such as global cerebral ischemia, neonatal hypoxia, and coronary artery bypass grafting (13, 26). And contribute to stroke patient recovery from neurologic deficits (27). The basis of neuroprotection may be due to that magnesium deficiency could be associated with the onset of an inflammatory response leading to increasing circulating levels of cytokines, which triggers oxidative responses in endothelial cells (28, 29). There is a statistically significant inverse association between magnesium intake and stroke risk (30, 31). However, the mechanism of neuroprotection remains unclear (32). Neuronal injury in stroke is caused by oxygen deficiency. Super physiological Mg has multiple potential pharmacology effects on stroke, and these effects result from a surge in the activation of interlinking pathophysiological pathways, with different pathways possibly predominating from the core and at the cusp of ischemic damage (33–35). Mg administration exhibits multiple potentially beneficial pharmacological effects in stroke. This element is a natural calcium channel blocker and has many metabolic effects in vivo. The peripheral administration of Mg enables the passage of the element through the intact blood-brain barrier. Thus, Mg can be used in the acute phase of stroke (18). Dietary Mg intake has been linked to a significant reduction in the risk of stroke in men and women (36). In an updated meta-analyses of prospective studies, the combined relative risk of total stroke was 0.87 (95% CI: 0.83, 0.92) for a 100 mg/day increase in Mg in-take, 0.91 (95% CI: 0.88, 0.94) for a 1000 mg/day increase in potassium intake, and 0.98 (95% CI: 0.94, 1.02) for a 300 mg/day increase in calcium intake (37).

### Mg deficiency and AD

AD is the most common form of dementia. This disorder is characterized by the progressive loss of neurons and synapses, mainly by high phosphorylation of tau and extracellular senile plaques composed of intracellular neurofibrillary tangle accumulation composed of β-amyloid protein composition (38–40). AD is a primary and degenerative brain disease with elusive pathogenesis. The pathologic changes are presented as brain atrophy and narrowing gyrus. Numerous senile plaques will be found in the cerebral cortical, and many neurofibrillary tangled neurons are observed in AD. The incidence of AD has positive correlation with age, and more female patients are afflicted with AD than male. Given the increasing life expectancy in developing countries, the number of people with dementia is expected to double every 20 yr, up from 115 million in 2050 (41). Furthermore, with the aging society, severe economic and social burdens from AD have drawn considerable attention. Increasing the concentration of Mg^2+^ in the extracellular fluid ([Mg^2+^]) results in a permanent increase in synaptic plasticity in the hippocampal neuronal network cultured in vitro to enhance learning and memory in experimental rats (4). Mg deficiency has been emerging as a risk factor for AD. The level of Mg diet is critical to maintain synaptic plasticity, and the decline in hippocampal synaptic connections has been associated with impaired memory (42). Cognitive decline is associated with the prevention of AD. In AD, factors related to the intake of Mg are particularly distinct. Several studies have suggested a neuroprotective action of Mg in the synaptic function (5). Decreased Mg level was found in various tissues of AD patients in clinical and laboratory studies (43,44). New findings in animal studies are promising and provide novel insights into the neuroprotective effects of Mg, and Mg treatment at the early stage may decrease the risk of cognitive decline in AD (45). Consequently, biomarkers of AD have become a hot spot in recent studies. The biomarkers of AD can be classified into two categories depending on the biological characteristics. The first category includes markers associated with Aβ deposition in the brain, such as the decrease of Aβ level in the cerebrospinal fluid. By contrast, the second category includes the degeneration of downstream neurons or damage-related biomarkers, mainly the cerebrospinal fluid tau protein, such as tau protein and phosphorylation of tau, which are conducive to diagnose the early AD patients without clinical symptoms (46). Based on its prediction effect of cognitive impairment and the role of Mg in AD, a persuasive trial is warranted to determine if Mg can be used as a biological marker.

### Mg deficiency and Parkinson’s disease

Parkinson’s disease is a common neurodegenerative disease that occurs in the substantial nigra and striatum (47). The substantia nigra is characterized by the presence of Lewy bodies containing threadiness α-synuclein (48). Magnesium interacts with α-synuclein, which inhibits α-Synuclein aggregation by immunoblotting (49). α-synuclein promoter is a susceptibility factor for idiopathic PD and it plays a key role in the path-ophysiology of PD. Early-onset PD has been linked to two point mutations in the gene that encodes α-synuclein, suggesting that disease may arise from accelerated fibrillization (48). An experiment showed the concentration of magnesium in the cortex, white matter, basal ganglia and brainstem of PD brain is low (50). The exact cause of its pathological changes is still not very clear, genetic, aging, oxidative stress, etc. Oxidative damage caused by magnesium deficiency is also reflected in PD (51). In Parkinson’s disease, magnesium levels are reduced (49). Parkinson’s patients may exhibit the following symptoms in the absence of magnesium: such as the first symptom of static tremor, caused by repeated forms of muscle contraction and relaxation; myotonicmyopathies; bradykinesia, that is, slowness of the movements, reduced movement range, reduced facial movements and abnormal posture & gait (48).

### Mg deficiency and Migraine

Migraine is a very common neurobiological headache disorder caused by increased excitability of the CNS (52). This condition is a very common clinical type of primary headache. Paroxysmal moderate to severe, pulsatile headache are the main manifestations of a migraine, which usually lasts for 4–72 h (53). Migraine can be accompanied by nausea and vomiting. Light and sound stimulation or daily activities can also aggravate headaches. However, the pathogenesis of migraine remains unclear. On the one hand, the vascular theory holds that intracranial vasoconstriction causes migraine premonitory symptoms, followed by extracranial and intracranial vascular tissue producing vasoactive peptides. This phenomenon results in aseptic inflammation leading to pulsatile headache. On the other hand, according to the theory of nerve, a change in the nerve function comes first in the attack of migraine, followed by a change in blood flow. The theory of peripheral pain mechanisms and trigeminal ganglion damage may be the neural basis of migraine. When trigeminal ganglion and its fibers are stimulated, the release of substance P, calcitonin gene-related peptide, and other neuropeptides can be increased. These active substances in adjacent brain blood vessels can cause vascular expansion and throbbing headache (54). Three approaches can lead to migraine caused by Mg deficiency. First, Mg deficiency can alter the release of neurotransmitter. Second, the platelets can become hyper aggregated. Third, cortical spreading depression is promoted (22). Therefore, magnesium can improve mitochondrial oxidative phosphorylation, 5-HT neurotransmission and the NO system during migraine (55). Serum Mg levels are significantly lower in migraine patients, and that the normal population is associated with the frequency of migraine attacks. These phenomena support the use of Mg in migraine prevention and treatment (56). In the case of magnesium sulfate and placebo control, the effect of magnesium sulfate is better (57). In order to determine whether patients with migraine have systemic magnesium deficiency, an oral magnesium load test is performed, and magnesium retention occurs in migraine patients, suggesting a generalized magnesium deficiency (58). Further study is necessary given that people with migraine are at an increased risk of ischemic stroke. This increased risk is only apparent in those who have migraine with aura and not in those with migraine without aura, and the relative risk will be doubled (59).

## Conclusion

If the body lacks magnesium or the concentration of magnesium ions is abnormal, it can cause a variety of neurological diseases. Previous review is known Mg has led us to the tentative that Mg plays a role in neurological diseases. Further summary of the disease’s pathogenesis, clinical manifestations and lack of Mg^2+^ have made in this review according to the recent literature. Meanwhile, according to the FDA’s standard, we listed food with high magnesium as an important reference to supplement magnesium more selective, as insufficient magnesium intake is the major cause of magnesium deficiency. Next, on the basis of magnesium deficiency as a causative factor of AD, proposed a new prospective that if we can research a new biomarker to detect the occurrence of AD.

## Ethical considerations

Ethical issues (Including plagiarism, informed consent, misconduct, data fabrication and/or falsification, double publication and/or submission, redundancy, etc.) have been completely observed by the authors.
